# Different Senses for Different Roles: Sexual Dimorphism in the Sensory System of a Scoliid Wasp

**DOI:** 10.3390/insects17020160

**Published:** 2026-02-02

**Authors:** Andrea Ferrari, Carlo Polidori

**Affiliations:** Department of Environmental Science and Policy (ESP), University of Milan, Via Celoria 26, 20133 Milan, Italy; andrea.ferrari@unimi.it

**Keywords:** scanning electron microscope, sensilla, ocellus, eye, antenna, *Scolia hirta*

## Abstract

Mating and feeding behavioural differences have been observed between male and female wasps (Hymenoptera: Aculeata). It is hypothesised that these behavioural differences are supported by morphological dimorphisms in their sensory systems. Here, this hypothesis was tested in the parasitoid aculeate wasp *Scolia hirta* (Scoliidae), which attacks beetle larvae located in the soil. We have found evidence of a link between the main stimuli used by the sexes to access their target resources and the morphology of the sensory system. Males have a more developed visual system with enlarged eyes, ocelli and a higher number of ommatidia, sinc they have to visually locate and chase females immediately after they emerge. Males possess nine types of sensilla (primarily hygrothermo- and mechanoreceptors), and females possess seven types of sensilla, primarily olfactory. In females, the sensilla placoidea occur in greater numbers and are larger in size, in line with their underground host-seeking behaviour. Furthermore, the females had a blunt tip on their distal flagellomere and wider fore tibiae, which suggests that they may use vibrational sounding to detect concealed hosts, similarly to what has been described for some non-aculeate parasitoid wasps.

## Simple Summary

The morphological dimorphism in the visual and antennal sensory systems of wasps has not yet been extensively investigated. However, it is interesting to understand whether the marked behavioural differences observed between males and females can also be linked to morphological differences. Therefore, the aim of this study is to provide the first quantitative description of sensory systems for the species *Scolia hirta*, a parasitic wasp whose females attack beetle larvae in the soil. It is hypothesised that males have better vision because they must chase females to mate, while females have a better sense of smell to find their hosts underground. The results confirm these hypotheses: males have proportionally larger compound eyes than females. Conversely, females have more olfactory sensilla on their antennae. In conclusion, the differences in behaviour are also reflected in the morphology of the sensory systems in this wasp species. Studies of this type are important for increasing our knowledge of the evolutionary connection between form and function in insects.

## 1. Introduction

The sensory system of insects integrates numerous sensory information from an assortment of visual, chemical or mechanical inputs [1]. These stimuli are present throughout an insect’s life, profoundly regulating their behaviour and determining the evolution of certain morphologies and adaptations to environmental conditions [1]. The visual and antennal sensory systems are important components of the insect sensory system, since they are involved in a plethora of tasks, for example, from vision to olfaction or mechanoreception.

The organs for photoreception typically consist in simple and compound eyes. Ocelli, which are simple eyes, are found in most aculeate Hymenoptera on the dorsal surface of the head (i.e., vertex) in a set of three; arranged at the corners of a triangle [1]. Due to the extensive convergence of ocellary cells and the supposed lack of image focusing, the ocelli are thought to be involved in light intensity perception rather than image formation [1]. Therefore, ocelli may act in concert with compound eyes to detect movement, to orient the body during fast locomotion [2], or to maximise light capture in crepuscular species [3]. Compound eyes are composed of facets called ommatidia, which contain pigment cells that prevent focused light from travelling outside the eye and therefore forming images [1]. Visual acuity and resolution can vary across species due to changes in eye size, the interommatidial angle, the number of ommatidia, or their density. It is thought that larger eyes, with a higher number of ommatidia and reduced interommatidial angles, are likely to increase visual resolution [4].

The organs responsible for mechano-, hygro-, thermal-, and chemical reception are the sensilla, which are found throughout the insect body, but especially in the final segments of the antennae. The basic structure of a sensillum includes a sensory neuron and cells that make up the shaft and socket, as well as providing protection for the neuron itself [1]. Sensilla are usually categorised according to their structure and associated function, but there is still no general agreement on this. However, the classification that includes the following types of sensilla is generally accepted: trichoid (olfactory, gustatory or mechanoreceptor function); placoid (olfactory function); basiconic (olfactory function); coeloconic/ampullacean (detect humidity); campaniform (hygrothermal-detecting function); and pit organ (hygrothermal-detecting function) [5,6,7,8]. It is thought that greater diversity of sensillar types and greater sensillar density likely increase chemical and mechanical sensitivity [9].

As evidenced in Aculeate Hymenoptera (bees, ants and stinging wasps), the antennal and visual sensory systems are under strong selective pressure since species with different biology (e.g., different diets, foraging strategies, or mating strategies) require different sensory equipment [10,11,12,13,14]. For example, in *Bombus* bees (Apidae), males of species that exhibit perching mating strategies are associated with larger eyes than those of species that exhibit patrolling behaviours [15]. In *Andrena* bees (Andrenidae), generalist species have more sensilla trichoidea than specialist species, suggesting that the sensillar equipment is likely to be under selective pressure related to flora recognition [16].

Sensory systems are also under sexual selection since males and females within the same species often differ in terms of their mate-finding or foraging behaviours [17]. These differences can be reflected in their sensory system morphology. For example, males of two *Megalopta* bee species (Halictidae) have relatively larger eyes compared to females, likely due to a continued dependence on vision for mate location [18]. Furthermore, an extreme example of sensory sex dimorphism in antennae is found in the Eucerini bee tribe (Apidae), where males have antennae that are two times longer than those of females and have many more pore plates and olfactory receptor neurons [19].

Nevertheless, most of the studies on the sensory systems of aculeate Hymenoptera are biassed towards bees (Apoidea), followed by social wasps (Vespidae) and ants (Formicidae) [20,21,22,23]. Studies on other lineages, especially those of aculeate parsitoids (e.g., Chrysididae, Mutillidae, Scoliidae), are still extremely scarce [24].

In general, for oviposition, female aculeate parasitoids rely heavily on chemical stimuli alone to locate host nests or hosts concealed in a substrate. This olfaction-driven search could also be aided by the perception of vibrations from the concealed host. Indeed, echolocation (e.g., vibrational sounding) have often evolved in response to the host remaining hidden under (or inside) a solid substrate in non-aculeate parasitoids [25]. However, this phenomenon is yet to be observed in aculeate parasitoids. Vibrational sounding is possible thanks to two morphological adaptations. These are the modified tips of the antennae of the female which have become blunt (like a hammer head), and the subgenual organs in the enlarged (swollen) female fore tibiae, which receive the vibrational information [26]. Conversely, males typically explore the area around where females emerge to find virgin females, using visual and chemical cues to do so. In aculeate parasitoids, the males then engage in scramble competitions to mate, as documented in Chrysididae [27,28,29] Mutillidae [30,31], and Scoliidae [32,33,34].

Here, we present the first study on the sexual dimorphism of the external visual and antennal sensory system in any scoliid wasp by investigating *Scolia hirta* (Schrank, 1781) (Hymenoptera: Scoliidae). Scoliid wasps (Hymenoptera: Scoliidae) are distributed worldwide, especially in the tropical and subtropical regions [35], and are larval parasitoids of various soil beetles (Coleoptera: Curculionidae, Scarabaeidae). Scoliid wasps are considered protandrous [36], with males able to detect females before they emerge as adults from the parasitised larvae underground. This results in scramble competition and the formation of the so-called “mating balls” where multiple males attempt to mate with a single female [31,37,38]. This localisation is thought to be mediated by olfactory cues (pheromones), suggesting that the male’s antennae are highly receptive to these volatile compounds [38]. Nevertheless, evidence from *Scolia affinis* Guérin-Méneville, 1830 also suggests that the female stops flying or lands when ready to mate, with the males following closely behind [39]. This would suggest that males should also have sharp visual acuity to locate and chase virgin females. After mating, it is thought that females start feeding on nectar and then start to search the soil for hosts. In other *Scolia* species, for example, females were observed flying in straight lines with periodic rapid turns at around 15 cm above the ground [40,41]. This would imply that females mostly rely on olfactory, rather than visual, cues to locate their hosts underground.

Through a quantitative morphological approach involving scanning electron microscopy, a set of functional traits related to visual acuity (e.g., compound eye size, median ocellus size, ommatidia density) and antennal perception (types and density of sensilla) were measured and compared between males and females of *S. hirta*. This study aims to provide the first detailed description of the sensilla types in scoliid wasps and to test the following hypotheses:

I. Males should invest more resources than females in vision due to their need to seek and chase virgin females [18,42].
II. Females should invest more resources than males in olfaction to locate their hosts concealed in the soil [24,43].
III. Given that the hosts are located in the soil, females may also use echolocation to detect beetle larvae, and hence, they should possess a blunt tip of the last flagellomere and swollen fore tibiae [25,44].

## 2. Materials and Methods

### 2.1. Studied Species

**Figure 1:**
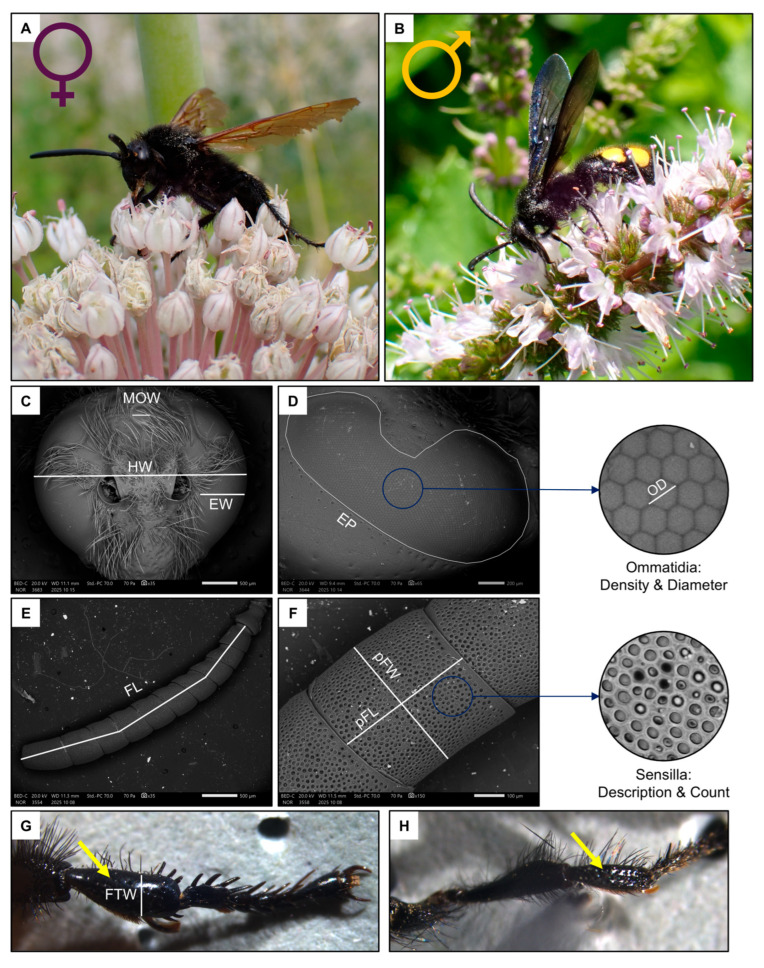
Schematic summary of the studied species, *Scolia hirta* ((**A**), female; (**B**), male) together with exemplificative images of the male head ((**C**), MOW: median ocellus width, HW: head width, EW: eye width), female compound eye ((**D**), EP: eye perimeter, OD: ommatidia diameter), female flagellum ((**E**), FL: flagellum length), female penultimate flagellomere ((**F**), pFW: penultimate flagellomere width, pFL: penultimate flagellomere length) and female fore tibia indicated by the yellow arrow ((**G**,**H**), FTW: fore tibia width of a female and male).

In Italy, the genus *Scolia* is the most specious genus of the tribe Scoliini (Hymenoptera: Scoliidae). *Scolia* has 7 species registered in Italy [45]. Of these species, *Scolia hirta* (Schrank, 1781) (Figure 1A,B) is one of the most widespread [45]. This species parasitises the larvae of *Cetonia* and *Protaetia* (Coleoptera: Scarabaeidae) [46].

For this study, a total of 10 females and 10 males, sampled in the Milan, Florence and Rome metropolitan areas (Italy) during the summers of 2023–2024, were analysed. Specimens were frozen upon collection, and then the intertegular distance (ITD), a proxy for body size [47], was measured from a dorsal image taken under a Leica M205 FCA stereomicroscope mounted with a LEICA DFC7000 T camera (LEICA, Wetzlar, Germany). After this, the front left leg was detached from the body, and the width of the tibia (FTW, Figure 1G) was measured under a stereomicroscope.

The head of the specimen was then cut off from the thorax and the antennae detached from the head with the forceps. Both heads and antennae were then mounted on aluminium stubs through an adhesive carbon tape, and then platinum-coated using a sputter (EM ACE600, LEICA, Wetzlar, Germany). One antenna was mounted ventrally and one dorsally, with the left and right ones being randomly shuffled.

### 2.2. Scanning Electron Microscopy

All samples were processed on the Scanning Electron Microscope (SEM) Zeiss LEO 1430 (Oberkochen, Germany) at the University of Milan. The SEM analysis was performed in high-vacuum (60–70 Pa) conditions, with resolution: 3.0 nm at 30 kV (secondary electrons), 10 nm at 3 kV (secondary electrons), and 4.0 nm at 30 kV (back-scattered electrons). The acceleration voltage was of 20–26 kV and the working distance was 9–12 mm.

**Figure 2:**
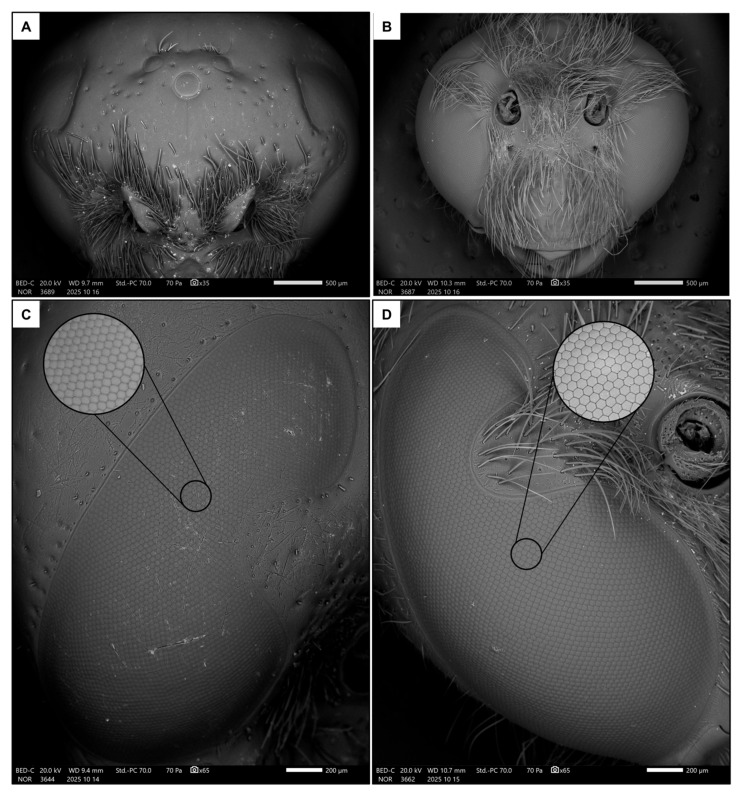
Frontal views of female (**A**) and male (**B**) heads, together with lateral views of eyes in female (**C**) and male (**D**). The focus shows a detail of the ommatidia.

Two images of the head were used to measure the traits related to the visual system (Figure 1C,D): one frontal and one lateral [48] (Figure 2A–D). Firstly, the width of the head (HW), the median ocellus (MOW) and the compound eye (EW, measured at its widest point, approximately below the antennal sockets) were measured from the frontal image (35× magnification).

Then, the eye perimeter (EP) was manually drawn and measured from the lateral image (50–65× magnification). The area of the eye was approximated to that of a spherical shell, which is calculated using the formula:

A = 2π*rh*

where *r* is the radius of the sphere and *h* is the height of the shell. This shows that the area is proportional to the perimeter of the base circle (2π*r*) multiplied by its height (*h*). Therefore, the area of the eye was estimated by multiplying the irregular, kidney-shaped perimeter of the eye (EP) by its height in the frontal view (EW) [13]. Given the significant curvature and irregularity of the eye, this is an approximate but plausible way of estimating the area of the compound eye.

A further magnification (200× magnification, Figure 2C,D) of the lateral portion of the compound eye was taken to measure the ommatidia average size (OD) as the mean of ten diameters (approximating the ommatidia as regular hexagons). Then, their density was calculated as their average number in three distinct square regions of interest (ROIs) of 100 µm-side. Knowing the average number of ommatidia in the ROIs and the area of the eye, the total number of ommatidia was estimated. Finally, the interommatidial angle was calculated as in [49]:

∆γ = √ (23’818/ommatidia number)

Next, the length of the flagellomere (FL) was measured, excluding the scape and pedicel, from an image of the whole antenna (35–40× magnification, Figure 1E). Then, the area of the penultimate flagellomere was estimated by multiplying its width (pFW) by its length (pFL) (Figure 1F).

**Figure 3:**
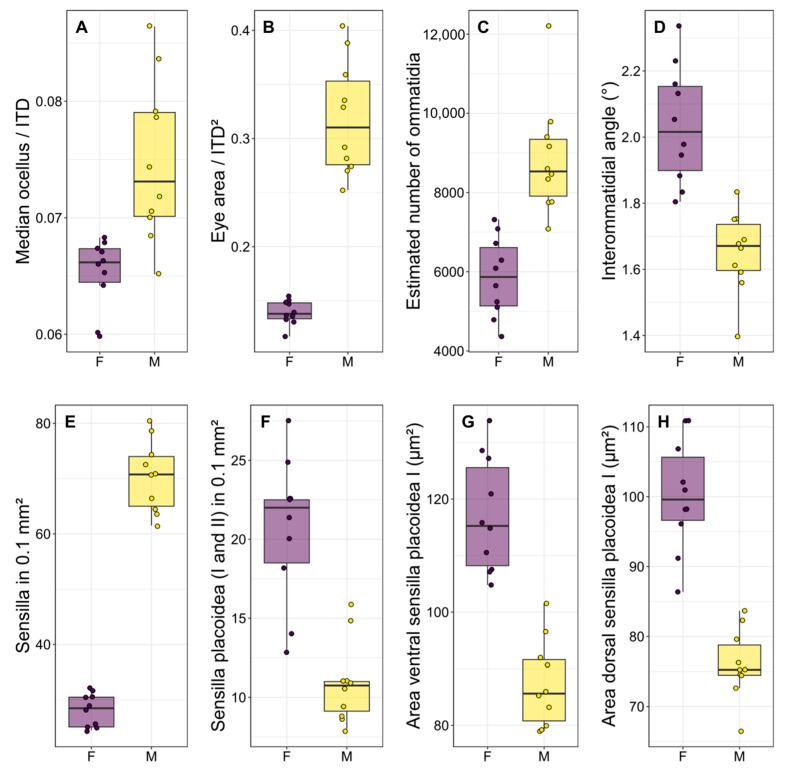
Boxplots illustrating the most significant differences (*p* < 0.001 for all comparisons) between the two sexes regarding some functional traits of the visual system (**A**–**D**) and sensilla (**E**–**H**). Each point is the measurement of an individual. F: females, M: males.

Images were taken of the penultimate flagellomere, the richest in sensilla in Hymenoptera [50], from both ventral and dorsal views (100–190× magnification, Figure 3C–F) for identification and count of sensilla types in a square ROI of 100 µm. Sensillar types were primarily classified based on external morphology following previous studies on aculeate Hymenoptera [24] as well as The Hymenoptera Anatomy Ontology (HAO) portal [51]. New types were added if they had not been described before. The main types of sensilla are the sensilla placoidea, s. coeloconica, s. basiconica, s. ampullacea and s. trichoidea. Some of them were further divided in subtypes. The area of one abundant type of s. placoidea was measured (N = 5, both on the ventral and on the dorsal side).

Finally, representative magnifications (700–3000× magnification) of all the sensilla were taken for illustrative purposes.

### 2.3. Statistical Analysis

Statistical analyses were run with base functions in R v.4.4.0 [52] through R-Studio, and all the images were processed in the free software ImageJ v.1.54i [53]. Plots were made with the R-packages *ggplot2* [54] using the colourblind-friendly *viridis* palette [55]. Correlations between functional traits were quantified with Spearman’s correlation coefficient. All data and the R-code are made freely available within the Supplementary Materials.

Ordinary (or generalised with Poisson distribution for counts) linear models were run to test the difference between sexes in (i) intertegular distance (ITD), (ii) head width, (iii) flagellum length, (iv) fore tibia width, (v) median ocellus size (weighted by ITD), (vi) compound eye (weighted by ITD^2^), (vii) ommatidia diameter, (viii) number of ommatidia, (ix) interommatidial angle, (x) density of ommatidia, (xi) penultimate flagellomere area (weighted by ITD), (xii) density of sensilla (total and separately for each of the more abundant types—dorsally or ventrally, depending on the type), and (xiii) area of one type of s. placoidea.

## 3. Results

### 3.1. General Dimorphism in Visual and Antennal Sensory System

There was a significant difference in body size between males and females, with females being larger than males in terms of both intertegular distance (*p* < 0.001, Table 1 and Table S1) and head width (*p* < 0.001, Table 1 and Table S1). There was a strong dimorphism in the macroscopic morphology of the eyes, which appear to occupy a significantly larger portion of the head in males than in females (Figure 2A,B), similarly to the median ocellus. Indeed, males had proportionally larger ocelli (Figure 3A, *p* < 0.001, Table 1 and Table S1) and eyes (Figure 3B, *p* < 0.001, Table 1 and Table S1). They also had more ommatidia (Figure 3C, *p* < 0.001, Table 1 and Table S1) and a smaller interommatidial angle (Figure 3D, *p* < 0.001, Table 1 and Table S1) than females. However, the size (*p* = 0.694, Table 1 and Table S1) and density (*p* = 0.878, Table 1 and Table S1) of ommatidia did not differ between the two sexes.

**Figure 4:**
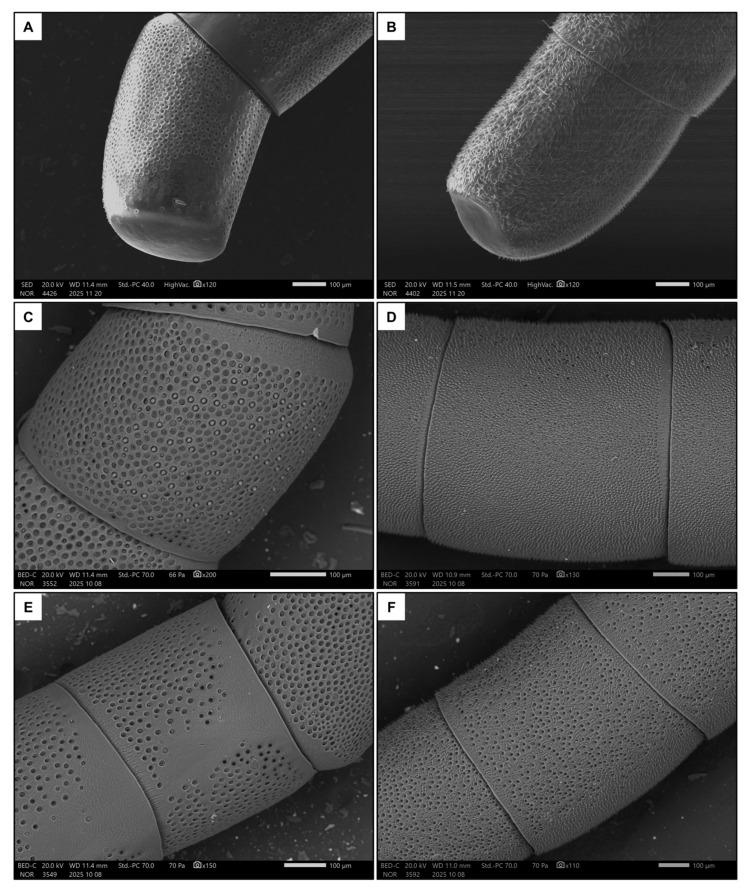
Examples of the last flagellomere in females (**A**) and males (**B**). Also shown are examples of the dorsal side of the penultimate flagellomere in females (**C**) and males (**D**), and the ventral side in females (**E**) and males (**F**).

Males had longer flagellum (*p* < 0.001, Table 1 and Table S1), due to having one additional flagellomere, and a larger penultimate flagellomere (*p* < 0.001, Table 1 and Table S1). There was a strong dimorphism between the macroscopic morphology of the last flagellomeres of females and males. In particular, the tip of the last flagellomere was much blunter in females (Figure 4A) than in males (Figure 4B). The dorsal side of the penultimate flagellomere was denser in sensilla (all types) in males (Figure 4D) than in females (Figure 4C). Females also had a medial ventral portion of the penultimate flagellomere devoid of sensilla (Figure 4E), whereas males had a denser concentration of sensilla (all types) (Figure 4F). In addition, females had proportionally larger fore tibiae than males (Figure 1, *p* < 0.001, Table 1 and Table S1).

### 3.2. Sexual Dimorphism in Antennal Sensilla Types and Density

**Figure 5:**
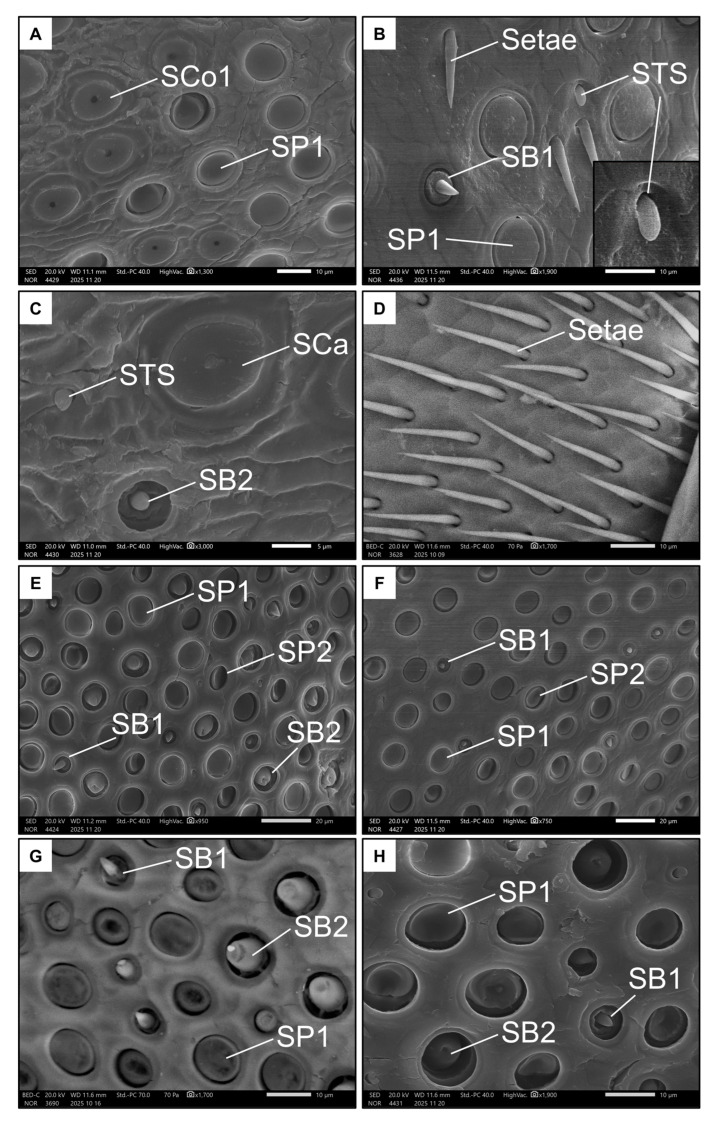
Examples of all the types of sensilla found in females on the ventral (**A**–**D**) and dorsal (**E**–**H**) side of the penultimate flagellomere. The images show Sensilla basiconica I (SB1), Sensilla basiconica II (SB2), Sensilla coeloconica I (SCo1), Sensilla ampullacea (SCa), Sensilla placoidea I (SP1), Sensilla placoidea II (SP2), and Sensilla “tongue-shaped” (STS).

Females had seven types of sensilla (Table 1 and Table 2). Six types were represented on the ventral side of the penultimate flagellomere. Sensilla placoidea I (SP1, Figure 5A,B) were by far the most prevalent. They have an almost circular shape and a flat surface (around 10 µm in diameter). In the distal portion of the flagellomere, the sensilla coeloconica I (SCo1, Figure 5A) were found. These were also circular and flat (with a diameter of around 10 µm), and with a distinctive pore in the middle. The rarer sensilla types were sensilla basiconica I (SB1, Figure 5B), which have a short, stout peg; sensilla basiconica II (SB2, Figure 5C), which have a sunken, stout peg (both long less than 5 µm); and sensilla ampullacea (SCa, Figure 5C), which have a large, spherical base of around 10 µm from which a small papilla protrudes.

Notably, a rare type of sensillum that differed in morphology from the already known types was also found on the ventral side of the penultimate flagellomere of females. These “tongue-shaped” sensilla (STS, Figure 5B) protrude from a pit and have a rather stout, knurled appearance. They lie on the surface of the antenna (i.e., they are not erected). Finally, the basal end of this flagellomere is dotted with long, thin bristles or setae (Figure 5D).

Only four types of sensilla were represented on the dorsal side of the penultimate flagellomere in females. SP1 (Figure 5F,G) was present again here, as well as SB1 and SB2, but at higher densities (Figure 5G, Table 1). The SB2 on this side of the antenna appeared larger than those on the ventral side, highlighting their large base and blunt tip. The seventh type of sensilla, the sensilla placoidea II (SP2, Figure 5E), are more elliptical and smaller (around 5 µm in diameter) than SP1. Interestingly, the surface of SP2 is inclined towards the inside of the pit.

**Figure 6:**
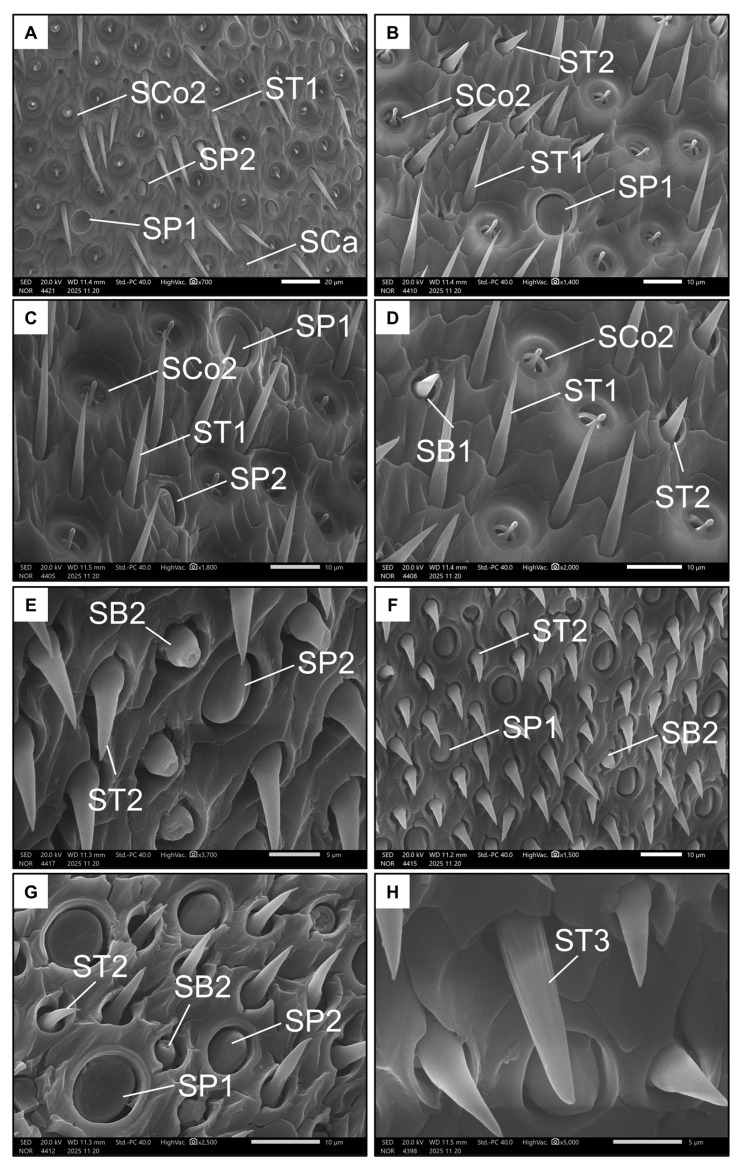
Examples of all the types of sensilla found in males on the ventral (**A**–**D**) and dorsal (**E**–**H**) side of the penultimate flagellomere. The images show Sensilla basiconica I (SB1), Sensilla basiconica II (SB2), Sensilla coeloconica II (SCo2), Sensilla ampullacea (SCa), Sensilla placoidea I (SP1), Sensilla placoidea II (SP2), Sensilla trichoidea I (ST1), Sensilla trichoidea II (ST2), and Sensilla trichoidea III (ST3).

Conversely, males had nine types of sensilla (Table 1 and Table 2). Seven types were represented on the ventral side of the penultimate flagellomere. Like females, males also exhibited SP1, SP2, SB1 and SCa (Figure 6A–D). Additionally, the ventral side of the penultimate flagellomere in males was rich in sensilla coeloconica II (SCo2, Figure 6D), which have a small, circular pit (less than 5 µm) with a rigid extruding peg that has a rounded tip. Males also had a high number of trichoid sensilla, including sensilla trichoidea I (ST1, Figure 6C,D), which are long and rigid with a spiral-like intertwined fibre structure, and sensilla trichoidea II (ST2, Figure 6D), which are shorter with a larger base and a smooth surface. Both ST types seemed non-porous.

Only five types of sensilla were present on the dorsal side of the penultimate flagellomere in males. SP1, SP2, and ST2 were also present on this side (Figure 6E–G). As with females, males have SB2 on the dorsal side of the penultimate flagellomere, but not on the ventral side. Finally, the ninth type of sensillum is the very rare sensillum trichoidea III (ST3 Figure 6H), which, like ST1, is long and rigid, but has a structure seemingly consisting of longitudinal, parallel fibres. Unlike ST1 and ST2, ST3 possess a single pore on their tips (Figure 6H).

In terms of the antennal sensory system, males had a higher density of sensilla (Figure 3E, *p* < 0.001, Table 2 and Table S1), due to the disproportionate number of sensilla trichoidea presented. However, males had a lower density of SP1 on both sides of the penultimate flagellomere (Figure 3F, *p* < 0.001, Table 2 and Table S1), as well as a lower density of SP2 on the ventral side (*p* < 0.001, Table 2 and Table S1), but not on the dorsal side of the penultimate flagellomere (*p* = 0.309, Table 2 and Table S1). Finally, males had smaller SP1 on both sides of the penultimate flagellomere (Figure 3G,H, *p* < 0.001, Table 2 and Table S1).

### 3.3. Covariation Between the Antennal Sensory System and Visual Systems

Some of these functional traits were found to covary between the antennal sensory system and visual systems, but very rarely. For instance, males with a higher density of ommatidia also exhibited fewer sensilla on the dorsal side of the penultimate flagellomere (Figure S1), and those with larger ommatidia had fewer SP1 on the dorsal side of the penultimate flagellomere (Figure S1). In addition, females with a higher density of ommatidia also exhibited fewer SB2 on the dorsal side of the penultimate flagellomere, but a higher number of ommatidia was also positively associated with more SP1 on the dorsal side of the penultimate flagellomere (Figure S2). Interestingly, the number of sensilla on the ventral side of the flagellomere was never found to covary with visual system traits in either sex.

## 4. Discussion

This is the first comprehensive, detailed study on the external visual and antennal sensory system, and the first quantitative comparison of these traits between males and females for any scoliid wasps. The results highlight novel morphological features such as the occurrence of two types of sensilla placoidea and of a previously undescribed sensillar type (STS, s. tongue-shaped). In addition, a striking sexual dimorphism is highlighted in this species spanning visual system, antennal morphology, sensillar equipment and even tibial morphology, which likely reflect the significant behavioural differences observed in males and females of parasitic aculeate Hymenoptera species.

### 4.1. Visual Sensory System

As in other aculeate Hymenoptera, *S. hirta* showed a strong sexual dimorphism in terms of visual system [11]. In particular, the ocelli of males were proportionally (compared to body size) larger, likely enabling them to react more sharply to changes in light intensity and therefore navigate more accurately in flight than females [56]. Additionally, males had proportionally larger eyes composed of more ommatidia arranged in a flatter configuration, as indicated by the lower interommatidial angle. Taken together, these functional traits strongly suggest that males of *S. hirta* have better visual resolution and increased light capture than conspecific females [56,57]. Interestingly, the size of the ommatidia was not different between males and females, and hence the density is also the same in a fixed area. In both sexes, facet size was also not correlated to body size, which confirms the substantial constancy of ommatidial size. This contrasts evidence from other aculeate Hymenoptera [15,24]. Since males have larger eyes, this means that the ommatidia facet is proportionally smaller (but with a higher number of ommatidia) in males than females. Albeit no solid conclusions may be drawn for this point, having more but smaller ommatidia may increase the image resolution, likely reflecting once again sharper vision in males [58].

These characteristics hint to the presence of a sex-biassed selective pressure, which is likely to be driven by the mating behaviour, as found in other aculeate Hymenoptera [59]. In fact, although studies on the reproductive behaviour of Scoliidae are still scarce [34,39], it seems that males engage in “scramble competition” to mate [33]. Few brief field observations during the sampling activity of the present study suggests that this also applies for *S. hirta*. This competition emerges when the high density of males makes territoriality unfavourable, as in bees [60]. In these cases, therefore, males must locate or chase virgin females as soon as possible using visual and chemical cues. This is because the first male to locate a virgin female will likely be the one to mate, as is the case of other parasitoid wasps such as *Nemka viduata* (Pallas, 1773) (Mutillidae) [31], or the bee *Colletes succinctus* (Linnaeus, 1758) (Colletidae) [61]. Therefore, the strong sexual dimorphism of the visual system found in these species may be driven by a selective pressure to efficiently locate virgin females. Indeed, the typical behaviour of Scoliidae females supports this differential pressure. *S. hirta* parasitises the underground larvae of at least two Scarabaeidae genera (Coleoptera), so they likely do not require sharp visual acuity, but rather a keen sense of smell [41].

Overall, the hypothesis (I) that *S. hirta* males should invest more resources in the visual system due to their need to find females is supported by our results. This example adds to the existing evidence for aculeate Hymenoptera [15,24,62,63], suggesting that locating flying virgin females, evolved in different phylogenetic lines, may represent a common driver of visual sensory system morphology. This further emphasises the phenotypic plasticity and adaptation of aculeate Hymenoptera in their response to behavioural such as mating or foraging strategies [12,64] or environmental pressures such as land use changes [48].

### 4.2. Antennal Sensory Sytem

The first notable finding is the small number of sensilla types on the penultimate flagellomere (less than ten in both sexes) in comparison with other aculeate Hymenoptera, such as bees and wasps [50,65]. Some morphological peculiarities emerged, but these are also difficult to compare with the more extensively described structures of other aculeate wasps that are more phylogenetically distant related. Indeed, Scoliidae are more closely related to ants and Apoidea than to other parasitic families, such as Mutillidae or Chrysididae [66].

Interestingly, *S. hirta* exhibited sensilla placoidea that were almost perfectly rounded, much more so than those found in other bees or wasps [16,50] and ants [67]. Rounded placoid sensilla are also likely present in the parasitoid genus *Cleptes* (Chrysididae) [67]. In addition, the size of this almost round sensilla placoidea (SP1) is similar to those found in Colletidae bees [5], albeit *S. hirta* is a larger species. The sensilla basiconica found in *S. hirta* closely resemble those pictured in the wasp *Ampulex* (Apoidea) and in the ant *Camponotus* [67], as well as in bees [68]. Additionally, the morphology of coeloconic and trichoid sensilla is similar to that found in other aculeates such as Mutillidae [24] or Philantidae [50,69].

It is interesting that females have a conspicuous portion of the ventral side of the penultimate flagellomere devoid of sensilla, and this deserves further investigation. There are cases of sensilla-free areas in the distal flagellomere of apoid wasps [50] but not on the penultimate flagellomere and not to the great extent found for *S. hirta*. Finally, of particular interest, an undescribed type of sensillum (“tongue-shaped”) was rarely observed protruding from a pit with a rather stout, knurled appearance. Located on the surface of the antenna, they seemingly lack any pores, suggesting an absence of olfactory or hygro-thermal receptor functions.

The antennae of females were surprisingly blunt, comparable with those of other parasitoid Hymenoptera that locate concealed hosts through vibrational sounding [24,44]. It is intriguing that females also possess swollen fore tibiae, likely confirming hypothesis (III), which hint at the possibility of enlarged subgenual organs [25]. However, the lack of field observations and precise knowledge of *Scolia* behaviour prevent us from drawing precise conclusions. In addition, it was shown that, through a series of dual-choice experiments, females of the scoliid wasp *Campsomeriella annulata* (Fabricius, 1793) perform frequent antennation on the soil, though at equal frequency with or without a buried host larva [70]. While this suggests that wasps would use vibrational sounding, in these experiments, the host larva was placed in a vinyl net bag of coarse mesh to prevent it from moving around, possibly altering the outcomes of the experiment. Further studies are necessary to confirm the preliminary hypothesis that *S. hirta* uses echolocation to assist host detection.

The observations that female slowly fly close to the ground in search of the hosts [40] is in accordance with the fact that females have more and larger sensilla placoidea, and more sensilla basiconica, compared to males. Sensilla placoidea and basiconica are olfactory discs, pore plates or pegs which may assist females in locating underground beetle larvae. Furthermore, the fact that sensilla placoidea and basiconica account for almost all the sensilla found in females (and are less abundant in males) suggests that, in females, more resources are invested in the olfactory system than males, confirming the hypothesis (II). This is consistent with other studies that have shown, for example, that large sensilla basiconica occur only on the antennae of female wasps [69]. Moreover, these results are also confirmed by what was found in other parasitic wasps, in which both the subtypes of sensilla placoidea showed higher abundance in females than males [71]. In addition, the scoliid wasp *C. annulata* searches for the host by using chemical stimuli emitted by larvae and faeces deposited in the soil [70], confirming our hypothesis (II) that females essentially rely on olfaction, rather than on vision, while searching for a host.

Finally, the strong sexual dimorphism was also highlighted by the fact that females completely lacked sensilla trichoidea, which are abundant and diverse (three subtypes) in males. While the function of these trichoid sensilla is unclear, ST1 and ST2 resemble other non-porous trichoid sensilla often associated with mechanoreception, similarly to what was found in Mutillidae [24] and Chalcidoidea [72]. Conversely, ST3 was uniporous and therefore probably had a taste function. As shown in other bees and wasps [73,74], males often touch and caress the antennae of females during the pre-copula. Therefore, these sensilla trichoidea may serve a mechanoreceptive (ST1 and ST2) and gustatory (ST3) function during mating. However, precise conclusions cannot be drawn due to the lack of ethological data on *Scolia* mating behaviour.

## 5. Conclusions

In conclusion, our hypothesis that males invest more in the visual system and females in the olfactory system is confirmed, and we suggest that females may also use echolocation to detect the hosts. This morphological dimorphism is consistent with much of the existing literature on other Hymenoptera, including other (mainly non-aculeate) parasitoids, and is also supported by the limited behavioural evidence on *S. hirta* and other previously studied scoliid wasps. This study encourages further research into the sensory system of aculeate Hymenoptera, a field which remains largely unexplored. Studying these morphological traits can be easily incorporated into the behavioural–ecological investigations on aculeate Hymenoptera, potentially providing evidence for still unappreciated links between morphology and function.

## Figures and Tables

**Table 1:** Summary of the statistics relating to the functional traits of the visual and antennal sensory systems measured in *S. hirta*. S: sensilla. Measures are given as mean ± standard deviation. The square ROI (100 × 100 µm) is the region for the count of all the sensilla types found. If only “present” is given, the density was too low to be assessed in that ROI.

Functional Trait	Females	Males
Intertegular distance (mm)	4.409 ± 0.357	3.605 ± 0.422
Head (mm)	3.949 ± 0.407	3.075 ± 0.253
Flagellum (mm)	3.638 ± 0.258	6.009 ± 0.472
Fore tibia width (mm)	0.707 ± 0.061	0.434 ± 0.056
Median ocellus (mm)	0.287 ± 0.024	0.269 ± 0.029
Eye (mm^2^)	2.728 ± 0.513	4.097 ± 0.731
Diameter of the ommatidia (mm)	0.024 ± 0.001	0.024 ± 0.001
Number of ommatidia	5863.803 ± 998.789	8857.546 ± 1435.704
Interommatidial angle (°)	2.035 ± 0.177	1.652 ± 0.122
Density of ommatidia (/µm^2^)	0.002 ± 0.001	0.002 ± 0.001
Penultimate flagellomere area (mm^2^)	0.211 ± 0.017	0.346 ± 0.050
Types of sensilla	7	9
Sensillae (total)	56.3 ± 5.9	140.7 ± 12.8
Sensillae (ventral)	20.2 ± 3.3	44.7 ± 8.7
Sensillae (dorsal)	36.1 ± 4.6	96.0 ± 15.0
SB1 (ventral)	0.9 ± 0.6	Present
SB1 (dorsal)	4.6 ± 1.9	Present
SB2 (ventral)	0.1 ± 0.3	Present
SB2 (dorsal)	7.4 ± 3.3	Present
SCo1 (ventral)	2.0 ± 2.5	-
SCo2 (ventral)	-	19.7 ± 4.5
SCa (ventral)	Present	0.1 ± 0.3
SP1 (ventral)	14.4 ± 3.1	2.3 ± 1.6
SP1 (dorsal)	16.0 ± 6.1	3.1 ± 2.3
SP2 (ventral)	2.8 ± 1.8	1.3 ± 2.1
SP2 (dorsal)	8.1 ± 2.2	15.2 ± 2.4
ST1 (ventral)	-	8.3 ± 5.4
ST2 (ventral)	-	13.0 ± 9.4
ST2 (dorsal)	-	77.2 ± 16.3
ST3 (dorsal)	-	0.5 ± 0.7
STS (ventral)	Present	-
Area SP1 ventral (µm^2^)	117.066 ± 10.131	87.342 ± 7.681
Area SP1 dorsal (µm^2^)	100.168 ± 7.979	76.049 ± 4.937

**Table 2:** Summary of the statistics relating to the functional traits of the visual and antennal sensory systems measured in *S. hirta*. S: sensilla. Measures are given as mean ± standard deviation. If only “present”—“absent” is given, the density was too low to be assessed in the 100 µm^2^ ROI.

Type of Sensilla	Supposed Function	Females	Males
Sensilla basiconica I—SB1	Olfaction	Ventral, Dorsal	Ventral
Sensilla basiconica II—SB2	Olfaction	Ventral, Dorsal	Dorsal
Sensilla coeloconica I—SCo1	Thermo-Hygroreception	Ventral	-
Sensilla coeloconica II—SCo2	Thermo-Hygroreception	-	Ventral
Sensilla ampullacea—SCa	Thermo-Hygroreception	Ventral	Ventral
Sensilla placoidea I—SP1	Olfaction	Ventral, Dorsal	Ventral, Dorsal
Sensilla placoidea II—SP2	Olfaction	Dorsal	Ventral, Dorsal
Sensilla trichoidea I—ST1	Mechanoreception	-	Ventral
Sensilla trichoidea II—ST2	Mechanoreception	-	Ventral, Dorsal
Sensilla trichoidea III—ST3	Mechanoreception	-	Dorsal
Sensilla “tongue-shaped”—STS	Unknown	Ventral	-

## Data Availability

The original contributions presented in this study are included in the article/Supplementary Materials. Further inquiries can be directed to the corresponding author.

## Supplementary Materials

The following supporting information can be downloaded at: https://www.mdpi.com/article/10.3390/insects17020160/s1, Table S1. Summary statistics of the ordinary and generalised linear models used to test the difference in the functional traits between the two sexes. ITD: intertegular distance, pFA: Penultimate flagellomere area, M: males, F: females, S: sensilla. Figure S1. Correlation matrix showing a scatterplot of the functional traits measured in males of S. *hirta* in the lower part and the corresponding Spearman’s correlation metrics in the upper part (only statistically significant, *p* < 0.05, are shown). Only the most abundant types of sensilla are shown. ITD: Intertegular distance; HW: head width; FL: flagellum length; FTW: fore tibia width; MOW: median ocellus width; EA: eye area; OD: ommatidia diameter; Num_O: number of ommatidia; Angle_O: interommatidial angle; Dens_O: density of ommatidia; pFA: area of the penultimate flagellomere; N_Sens: all the sensilla counted on both sides of the pFA; Sens_V: all the sensilla counted on the ventral side of the pFA; Sens_D: all the sensilla counted on the dorsal side of the pFA; SCo2_V: sensilla coeloconica type II counted on the ventral side of the pFA; SCa_V: sensilla ampullacea counted on the ventral side of the pFA; SP1_V: sensilla placoidea type I counted on the ventral side of the pFA; SP1_D: sensilla placoidea type I counted on the dorsal side of the pFA; SP2_V: sensilla placoidea type II counted on the ventral side of the pFA; SP2_D: sensilla placoidea type II counted on the dorsal side of the pFA; ST1_V: sensilla trichoidea type I counted on the ventral side of the pFA; ST2_V: sensilla trichoidea type II counted on the ventral side of the pFA; ST2_D: sensilla trichoidea type II counted on the dorsal side of the pFA; A_SP1_V: area of the sensilla placoidea type I on the ventral side of the pFA; A_SP1_D: area of the sensilla placoidea type I on the dorsal side of the pFA. Figure S2. Correlation matrix showing a scatterplot of the functional traits measured in males of S. hirta in the lower part and the corresponding Spearman’s correlation metrics in the upper part (only statistically significant, *p* < 0.05, are shown). Only the most abundant types of sensilla are shown. ITD: Intertegular distance; HW: head width; FL: flagellum length; FTW: fore tibia width; MOW: median ocellus width; EA: eye area; OD: ommatidia diameter; Num_O: number of ommatidia; Angle_O: interommatidial angle; Dens_O: density of ommatidia; pFA: area of the penultimate flagellomere; N_Sens: all the sensilla counted on both sides of the pFA; Sens_V: all the sensilla counted on the ventral side of the pFA; Sens_D: all the sensilla counted on the dorsal side of the pFA; SB1_D: sensilla basiconica type I counted on the dorsal side of the pFA; SB2_D: sensilla basiconica type II counted on the dorsal side of the pFA; SCo1_V: sensilla coeloconica type I counted on the ventral side of the pFA; SP1_V: sensilla placoidea type I counted on the ventral side of the pFA; SP1_D: sensilla placoidea type I counted on the dorsal side of the pFA; SP2_D: sensilla placoidea type II counted on the dorsal side of the pFA; STS_V: sensilla “tongue-shaped” counted on the ventral side of the pFA; A_SP1_V: area of the sensilla placoidea type I on the ventral side of the pFA; and A_SP1_D: area of the sensilla placoidea type I on the dorsal side of the pFA.

## Abbreviations

The following abbreviations are used in this manuscript:

ITD	Intertegular distance
HW	Head width
FL	Flagellum length
FTW	Fore tibia width
MOC	Median ocellus
EW	Eye width (frontal view)
EP	Eye perimeter (lateral view)
EA	Eye area; calculated as shown in the text
OD	Ommatidia density
SB1	Sensilla basiconica I
SB2	Sensilla basiconica II
SCo1	Sensilla coeloconica I
SCo2	Sensilla coeloconica II
SCa	Sensilla ampullacea
SP1	Sensilla placoidea I
SP2	Sensilla placoidea II
ST1	Sensilla trichoidea I
ST2	Sensilla trichoidea II
ST3	Sensilla trichoidea III
STS	Sensilla “tongue-shaped”
