# Supplementary material for: Different Senses for Different Roles: Sexual Dimorphism in the Sensory System of a Scoliid Wasp
Source: Insects. 2026 Feb 2;17(2):160. doi: 10.3390/insects17020160 (PMC12940552; doi:10.3390/insects17020160)
Supplement: Supplementary file 1 [file insects-17-00160-s001.zip › Ferrari_Polidori_Supplementary_Information-Rev1.pdf]

Supporting information of the ms:

Different senses for different roles: sexual dimorphism in the sensory system of a scoliid wasp

Andrea Ferrari [1] and Carlo Polidori [1] *

[1] Department of Environmental Science and Policy (ESP), University of Milan, via Celoria 26, 20133, Milan (Italy)

* Correspondence: carlo.polidori@unimi.it

Table S1. Summary statistics of the ordinary and generalised linear models used to test the difference in the functional traits between the two sexes. ITD: intertegular distance, pFA: Penultimate flagellomere area, M: males, F: females, S: sensilla.

Functional trait	Estimate	t (M *vs* F)	P-Value
Intertegular distance	-0.804	-4.603	**<0.001**
Head width	-0.874	-5.771	**<0.001**
Flagellomere length / ITD	0.849	25.610	**<0.001**
Fore tibia width / ITD	-0.040	-14.100	**<0.001**
Median ocellus / ITD	0.010	4.010	**<0.001**
Eye area / ITD^2	0.179	10.530	**<0.001**
Ommatidia diameter	0.001	0.400	0.694
Number of ommatidia	0.412	77.480	**<0.001**
Interommatidial angle	-0.382	-5.626	**<0.001**
Density of ommatidia	0.001	0.154	0.879
Penultimate flagellomere area / ITD^2	0.016	16.480	**<0.001**
Density of sensilla	74.060	4.912	**<0.001**
Density of sensilla placoidea I (dorsal)	-32.911	-6.887	**<0.001**
Density of sensilla placoidea I (ventral)	-31.029	-10.230	**<0.001**
Density of sensilla placoidea II (dorsal)	2.774	1.047	0.309
Density of sensilla placoidea II (ventral)	-4.659	-2.603	**0.018**
Area of sensilla placoidea I (dorsal)	-24.119	-8.128	**<0.001**
Area of sensilla placoidea I (ventral)	-29.724	-7.393	**<0.001**

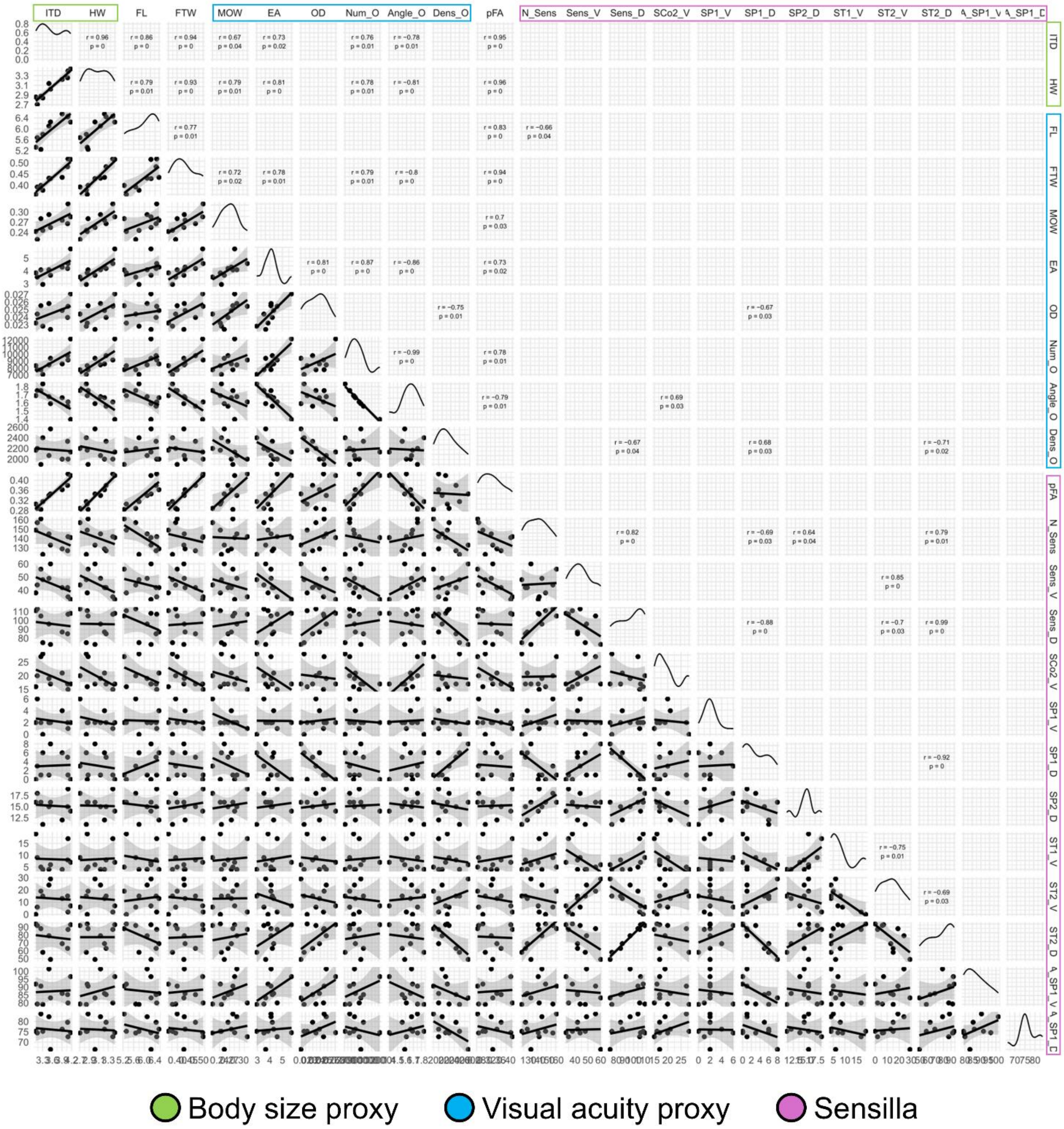

Figure S1. Correlation matrix showing a scatterplot of the functional traits measured in males of S. *hirta* in the lower part and the corresponding Spearman's correlation metrics in the upper part (only statistically significant, $P < 0.05$, are shown). Only the most abundant types of sensilla are shown. ITD: Intertegular distance, HW: Head width, FL: Flagellum length, FTW: Fore tibia width, MOW: Median ocellus width, EA: Eye area, OD: Ommatidia diameter, Num_O: Number of ommatidia, Angle_O: Interommatidial angle, Dens_O: Density of ommatidia, pFA: Area of the penultimate flagellomere, N_Sens: All the sensilla counted on both sides of the pFA, Sens_V: All the sensilla counted on the ventral side of the pFA, Sens_D: All the sensilla counted on the dorsal side of the pFA, SCo2_V: Sensilla coeloconica type II counted on the ventral side of the pFA, SCa_V: Sensilla ampullacea counted on the ventral side of the pFA, SP1_V: Sensilla placoidea type I counted on the ventral side of the pFA, SP1_D: Sensilla placoidea type I counted on the dorsal side of the pFA, SP2_V: Sensilla placoidea type II counted on the ventral side of the pFA, SP2_D: Sensilla placoidea type II counted on the dorsal side of the pFA, ST1_V: Sensilla trichoidea type I counted on the ventral side of the pFA, ST2_V: Sensilla trichoidea type II counted on the ventral side of the pFA, ST2_D: Sensilla trichoidea type II counted on the dorsal side of the pFA, A_SP1_V: Area of the sensilla placoidea type I on the ventral side of the pFA, A_SP1_D: Area of the sensilla placoidea type I on the dorsal side of the pFA.

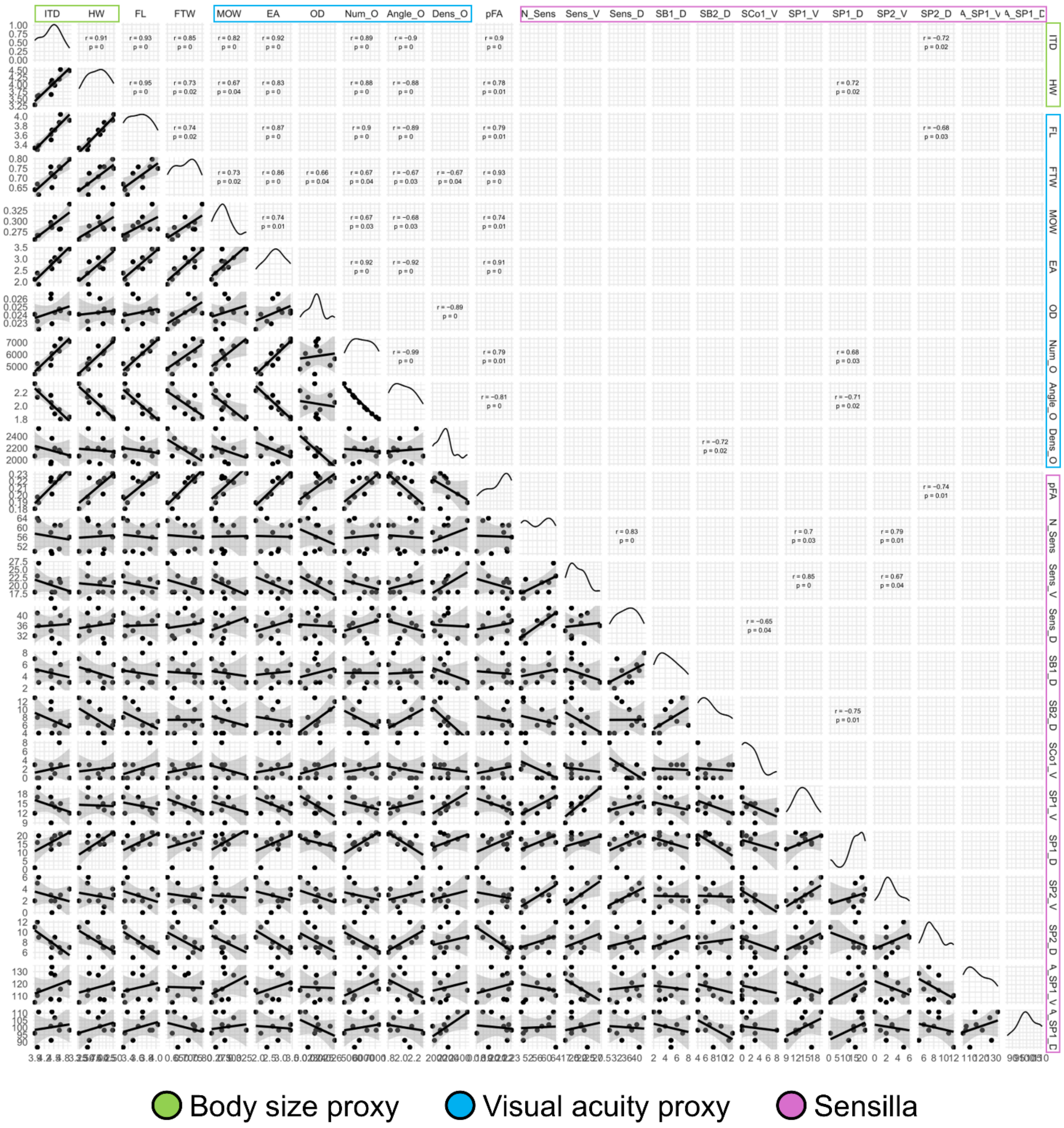

Figure S2. Correlation matrix showing a scatterplot of the functional traits measured in males of S. *hirta* in the lower part and the corresponding Spearman's correlation metrics in the upper part (only statistically significant, P < 0.05, are shown). Only the most abundant types of sensilla are shown. ITD: Intertegular distance, HW: Head width, FL: Flagellum length, FTW: Fore tibia width, MOW: Median ocellus width, EA: Eye area, OD: Ommatidia diameter, Num_O: Number of ommatidia, Angle_O: Interommatidial angle, Dens_O: Density of ommatidia, pFA: Area of the penultimate flagellomere, N_Sens: All the sensilla counted on both sides of the pFA, Sens_V: All the sensilla counted on the ventral side of the pFA, Sens_D: All the sensilla counted on the dorsal side of the pFA, SB1_D: Sensilla basiconica type I counted on the dorsal side of the pFA, SB2_D: Sensilla basiconica type II counted on the dorsal side of the pFA, SCo1_V: Sensilla coeloconica type I counted on the ventral side of the pFA, SP1_V: Sensilla placoidea type I counted on the ventral side of the pFA, SP1_D: Sensilla placoidea type I counted on the dorsal side of the pFA, SP2_D: Sensilla placoidea type II counted on the dorsal side of the pFA, STS_V: Sensilla "tongue-shaped" counted on the ventral side of the pFA, A_SP1_V: Area of the sensilla placoidea type I on the ventral side of the pFA, A_SP1_D: Area of the sensilla placoidea type I on the dorsal side of the pFA.
